# End-to-end self-assembly of gold nanorods in isopropanol solution: experimental and theoretical studies

**DOI:** 10.1007/s11051-015-3285-x

**Published:** 2015-12-11

**Authors:** M. Gordel, K. Piela, R. Kołkowski, T. Koźlecki, M. Buckle, M. Samoć

**Affiliations:** Advanced Materials Engineering and Modelling Group, Faculty of Chemistry, Wrocław University of Technology, WybrzeżeWyspiańskiego 27, 50-370 Wroclaw, Poland; Laboratoire de Biologie et Pharmacologie Appliquée, CNRS, École Normale Supérieure de Cachan, Avenue du Président Wilson 61, 94230 Cachan, France; Department of Physical and Quantum Chemistry, Wrocław University of Technology, Wybrzeże Wyspiańskiego 27, 50-370 Wroclaw, Poland; Laboratoire de Photonique Quantique et Moléculaire, CNRS, École Normale Supérieure de Cachan, Avenue du Président Wilson 61, 94230 Cachan, France; Department of Chemical Engineering, Faculty of Chemistry, Wrocław University of Technology, Wyspiańskiego 27, 50-370 Wrocław, Poland

**Keywords:** Gold nanorods, End-to-end assembly, Local electric field enhancement, Plasmon modes

## Abstract

**Abstract:**

We describe here a modification of properties of colloidal gold nanorods (NRs) resulting from the chemical treatment used to carry out their transfer into isopropanol (IPA) solution. The NRs acquire a tendency to attach one to another by their ends (end-to-end assembly). We focus on the investigation of the change in position and shape of the longitudinal surface plasmon (l-SPR) band after self-assembly. The experimental results are supported by a theoretical calculation, which rationalizes the dramatic change in optical properties when the NRs are positioned end-to-end at short distances. The detailed spectroscopic characterization performed at the consecutive stages of transfer of the NRs from water into IPA solution revealed the features of the interaction between the polymers used as ligands and their contribution to the final stage, when the NRs were dispersed in IPA solution. The efficient method of aligning the NRs detailed here may facilitate applications of the self-assembled NRs as building blocks for optical materials and biological sensing.

**Graphical Abstract:**

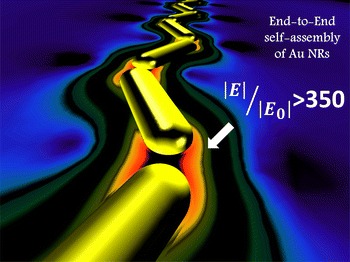

## Introduction

Plasmonic nanoparticles made of noble metals are promising as materials since they possess unique properties due to the presence of localized surface plasmon resonances that lead to enhanced scattering and absorption of light. Such nanoparticles may possess tunable optical properties, for example, gold nanorods (NRs) have two prominent absorption bands, a short wavelength band due to transverse plasmon excitation located around 520 nm and a long wavelength band caused by longitudinal plasmons and which can be tuned to be in either the visible or NIR regions, depending on the NRs aspect ratio (Liu et al. 2014). Additional modifications of the surface plasmon bands occur when the NRs are assembled close together, i.e., end-to-end or side-by-side. A red shift of the longitudinal surface plasmon (l-SPR) band is observed when the NRs are linked end-to-end; however, side-by-side linking of the NRs causes a red shift of the transverse surface plasmon resonance (t-SPR) band and a blue shift of the l-SPR band (Jain et al. 2006). The self-assembly of the NRs is possible because of the anisotropic shape of the nanoparticles and narrow size distribution.

Recently, increasing attention has been directed toward the assembly of metal nanoparticles, typically fabricated using wet chemistry methods, into two- and three-dimensional order structures (Mahmoud 2014; Yang et al. 2015), for use in biodiagnostics (Stewart et al. 2014), optical switching (Kang et al. 2013; Peroukidis et al. 2014), photonic waveguiding (Bernal Arango et al. 2012), or optoelectronics (Rakic et al. 1998). A crucial requirement toward the successful integration of such plasmonic nanoparticles into functional nanodevices using various liquid phase approaches is the need for an efficient and reproducible way of transferring them into solvents with a different polarity. So far, there have been well-described biological molecule-based pathways of assembling NRs where the process took place in water. The linking schemes involved proteins (Hamon et al. 2014; Wang and Tang 2014), streptavidin–biotin interactions (Caswell et al. 2003; Gole and Murphy 2005), DNA with thiol groups (Dujardin et al. 2001; Shanmugam et al. 2014; Thomas et al. 2004), or co-operative hydrogen-bonding schemes based on thiolated molecules (Thomas et al. 2004). However, all these procedures require additional stages of time-consuming functionalization as well as careful control of pH and salt concentration. In addition, most assemblies have rather poor stability and can only exist in water since, for example, proteins denaturize in organic solvents.

The most stable interactions that gold atoms can establish are with sulfur-containing ligands, i.e., ones with thiol, disulfide, and thiolate groups (Daniel and Astruc 2004). However, non-covalent interactions such as electrostatic interactions or physical adsorption can also be of use in NRs assembly processes and they offer the benefit of time saving and reduction in the complexity of ligand preparation. The relative simplicity of approaches using non-covalent interactions is also an advantage over complex covalent immobilization or specific recognition methods. The energy of covalent interactions is on the order of 150–450 kJ mol^−1^ for single bonds, non-covalent interactions are usually considerably weaker, although this depends on their type, e.g., ion–ion (200–300 kJ mol^−1^), dipole–dipole (5–50 kJ mol^−1^), ion–dipole (50–200 kJ mol^−1^), hydrogen bonding (4–120 kJ mol^−1^), van der Waals (<5 kJ mol^−1^), and hydrophobic interaction related to solvent–solvent interaction energy (<10 kJ mol^−1^) (Steed et al. 2007). Strong non-covalent interactions can be used to produce a modification of the surface of the NRs that prevents their aggregation.

We have characterized some optical properties of plasmonic nanostructures (Gordel et al. 2014; Olesiak-Banska et al. 2012), notably their non-linear absorption, in aqueous environment; however, an important challenge is to find an easy way of transferring such structures into an organic solvent. We describe in this paper such a process where, after the completion of the transfer, the nanoparticles are found to be already self-assembled in a certain way. We explore here the properties of end-to-end self-assembled nanostructures composed of NRs in isopropanol solution (IPA) and we discuss the obtained experimental and theoretical results. Precise spectroscopic characterization of the NRs solution at each stage of the transfer procedure was performed in the UV–Vis–NIR range and the results provided information about the interactions taking place between the gold surface and the ligands. We also discuss the possible mechanism of NRs end-to-end assembly in IPA solution. Additionally, we have theoretically modeled the interactions, providing an explanation for the l-SPR band being broader and red-shifted when the NRs are transferred into IPA solvent. The hot-spots created between the NRs ends and seen in the maps of the electromagnetic field enhancement may have a considerable potential in plasmon-enhanced Raman spectroscopy (Wei and Xu 2013).

## Experimental

### Gold nanorods synthesis

The NRs were synthesized by the procedure reported previously (Gordel et al. 2014; Sau and Murphy 2004). Briefly, first a solution of the seed nanoparticles (3.5 nm) was prepared using reduction of a gold salt (gold(III) chloride trihydrate) by borohydride (reduction of Au^3+^ to Au^0^) with hexadecyltrimethylammonium bromide (CTAB) used as a capping agent. This was followed by the growth procedure where gold salt is reduced by ascorbic acid in the presence of the CTAB micelles. Additionally, silver nitrate was added to the reaction mixture, at a concentration suitable for the appropriate aspect ratio of gold nanorods. The reaction was performed at 29 °C. To arrest the shortening of the nanorods, 15 mL of 1 mM Na_2_S solution was added into the growth solution one hour after seed injection, and the solution was left for 15 min in water bath at 29 °C (Gordel et al. 2014). Then, the growth solution was transferred into centrifuge tubes and centrifuged twice at 14,000 rpm for 20 min. Finally, the solution was re-suspended in 5 mL of water (NRs@CTAB).

### Transfer of gold nanorods into isopropyl alcohol (IPA)

The NRs transfer into IPA was based on the procedure reported in Ref. (Pastoriza-Santos et al. 2006), implemented with some modifications. First, 5 mL of NRs solution obtained after the synthesis was added dropwise to 5 mL of polystyrene sulphonate (PSS) (Mw 70,000 2 g/L, 6 mM NaCl, aqueous solution), sonicated previously for 30 min. The solution was kept under stirring for 3 h, then centrifuged twice at 1000 rpm for 25 min, and dispersed in 5 mL of water (NRs@PSS). Next, the solution obtained in the previous step was added into 5 mL of poly-(allylamine hydrochloride) (PAH) (Mw 17,500 2 g/L, 6 mM NaCl, aqueous solution). Stirring was continued for 3 h and the solution was centrifuged twice at 1000 rpm for 25 min (NRs@PAH). 5 mL of PSS/PAH coated NRs was vortexed overnight with 5 mL of poly(vinylpyrrolidone) (PVP) (Mw 10,000, 4 g/L, aqueous solution). After centrifugation, the final pellet was dispersed in 1.5 mL of IPA (NRs@PVP).

### Electrokinetic potential

Electrokinetic potentials have been measured by means of electrophoretic light scattering, using a Zetasizer 2000 apparatus (Malvern Instruments). Immediately before the measurement, dispersions of nanorods were diluted with an appropriate isotonic phosphate-buffered saline (PBS). The buffers were prepared according to Sorensen (Ghosh and Jasti 2005), using analytical grade NaH_2_PO_4_, Na_2_HPO_4_, NaCl, and ASTM Type I deionized water (D^1193^ 2011), then filtered through 0.45 μm membrane. Measurements were carried out at 25.0 ± 0.1 °C, using the rapid mode. The value of zeta potential reported for each sample corresponds to the average of five measurements.

### Sample characterization

The sizes of the NRs as well as their arrangement were characterized by a FEI Tecnai G2 20 X-TWIN Transmission Electron Microscope. The solutions were dropped onto TEM grids, dried, and then measured. The absorption spectra of the solutions were determined with a JASCO V-670 spectrophotometer.

The attenuated total reflection (ATR) spectra of commercially available powdered CTAB, PSS, PAH, and PVP, taken without further purification, were obtained by fixing on the diamond crystal surface, and the measurements were carried out in the 370–4000 cm^−1^ range with 4 cm^−1^ resolution. NRs@CTAB, NRs@PSS, NRs@PAH, and NRs@PVP solutions were added dropwise on diamond crystal, evacuated, and ATR spectra were measured in the same range and with the same spectral resolution. In the case of very low concentration, several layers of solution were put on the diamond crystal before measurements. Additionally, ATR spectra of NRs suspended in a different IPA to H_2_O ratio were measured under the same conditions. All spectra were collected under vacuum (<1 hPa) on a Bruker Vertex70v FT–IR spectrometer with accumulation of 64 scans. The ATR spectra were transformed into absorbance spectra and then normalized using OPUS software. The approximate percentage content of surfactant and each polymer in the experimental final stage was estimated on the basis of isolated intensity of the most characteristic band (20 % CTAB, 15 % PSS, 20 % PAH, and 45 % PVP).

### Numerical simulations

Plasmonic properties of NR assemblies were modeled using finite element method implemented in COMSOL Multiphysics software. Maxwell equations were solved in the frequency domain, employing spherical perfectly matched layers and a scattering boundary condition. Extinction cross section was obtained by adding absorption and scattering cross sections, calculated according to the formulas given by Knight and Halas (Knight and Halas 2008). NRs were modeled as gold cylinders with half-spherical tips, of total length 32.1 nm and diameter 9.4 nm, enclosed by a 0.5 nm-thick Na_2_S layer (assumed refractive index 1.55). Optical properties of gold were based on experimental data obtained by Johnson and Christy (Johnson and Christy 1972). Refractive index of a layer surrounding the NRs was calculated as a weighted average based on the relative content of the mixture of polymers and surfactants: 45 % PVP (*n* = 1.4215), 15 % PSS (*n* = 1.3718), 20 % PAH (*n* = 1.382), and 20 % CTAB (*n* = 1.449) (based on the ATR spectrum). The thickness of this layer, enclosing the NRs assembly, was in the range of 5–15 nm. The outermost surrounding medium was IPA with refractive index *n* = 1.3776. We investigated a single NR, as well as quasi-linear chains of two, three, and four NRs, with 1.5 nm gap between the tips. The incident electric field was defined as a uniform, normally incident plane wave, polarized either parallel or perpendicular to the axis of the chain. NRs within the chain were oriented either at +15° or at −15° with respect to the chain axis, that is either ±15° or ±75° with respect to the incident polarization.

In our electrodynamic simulations, the shortest distance between metallic surfaces of two neighboring nanoparticles was set to 1.5 nm, corresponding to an average distance measured in TEM images and including two Na_2_S layers of thickness 0.5 nm, separated by a 0.5 nm gap of polymer-surfactant mixture. Although such distance might be considered very short compared to a typical length scale of plasmonic effects modeled using classical electrodynamic approaches, the classical description remains still valid in this case. Non-locality and retardation of the response of free electron gas (David and De Abajo 2011) as well as the effect of quantum tunneling (Esteban et al. 2012; Teperik et al. 2013) have recently been found to cause considerable shifts of plasmon resonance bands and to diminish the electric field enhancement in the gap region of nanoparticle dimers. However, all these phenomena become significant only for sub-nanometer gaps, depending also on the local surface curvature. In our case, the curvature diameter of nanorod tips is on the order of 10 nm, while the Na_2_S layer prevents the two metallic surfaces from approaching each other to a distance shorter than 1 nm. By comparison with recently investigated case of gold nanosphere dimers (David and De Abajo 2011), the frequency shifts caused by electron gas non-locality can be neglected in this regime, see Fig. 5b in Ref. (David and De Abajo 2011). Similarly, no meaningful electron transfer between the metallic nanoparticles can occur for distances >0.5 nm (Esteban et al. 2012; Teperik et al. 2013), which has been demonstrated on the example of sodium nanosphere and nanowire dimers (about 4.3 and 10 nm in diameter, respectively) by showing that the full quantum mechanical modeling is equivalent to the classical description in this regime. Taking into account the above findings, the results obtained using the Comsol package and presented in this article can be considered trustworthy, although we should admit that a hypothetical plasmonic system of the same structure but having 3–4 times smaller dimensions would already require some corrections involving non-local and quantum effects.

## Results and discussion

### End-to-end assembly of gold nanorods

**Fig. 1:**
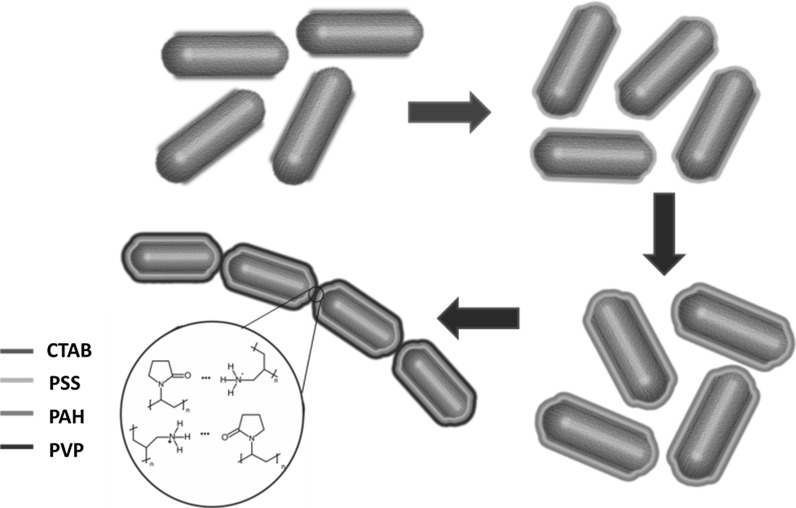
Cartoon of layer-by-layer deposition of polymers via electrostatic interactions onto the NRs

The transfer of NRs from water into isopropanol is based on multi-step surface-modifying reactions. In water, the NRs have a positively charged surface due to CTA^+^ ions strongly absorbed at the side surfaces (Yu and Irudayaraj 2007). The electrostatic repulsion between the NRs is responsible for their stability in solution. The transfer procedure implemented by us is a Layer-by-Layer Method (Hammond 2004) in which the gold surface is tightly covered by polymers of alternating charge. At first PSS (a negatively charged polymer) is used to modify the surface by tightly covering the positive charge layer given by CTA^+^ ions by one with the negative charge due to ${\text{SO}}_{3}^{ - }$ ionic groups. Most of the CTAB molecules are supposed to be adsorbed on the sides of the NRs (Yu and Irudayaraj 2007), then negatively charged PSS covers surface of the NRs (Fig. 1). Further procedure involves reactions with PAH and PVP, which additionally stabilize the nanoparticles in IPA solvent. PVP forms hydrogen bonds with water, but since the NPs are transferred into IPA solvent, this interaction no longer exists. Instead, hydrogen bonds are created between carbonyl and amino groups from PVP and PAH, respectively, which has a significant impact on the self-assembly.

**Fig. 2:**
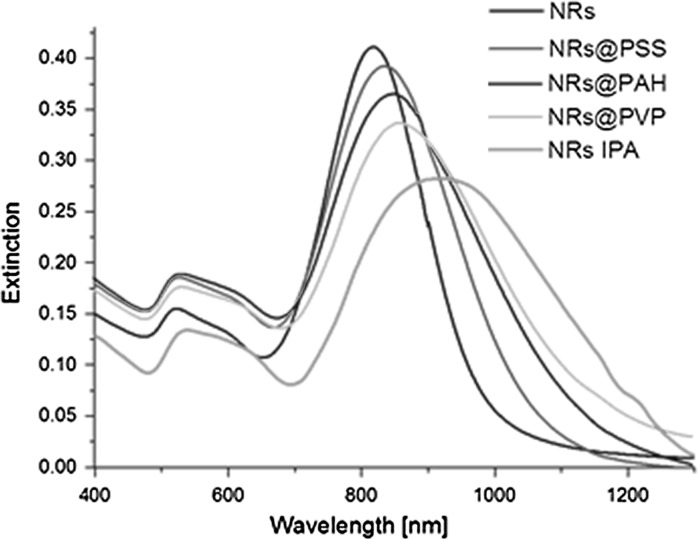
Extinction spectra of NRs suspended in water (*black line*), covered by PSS (*red line*), PAH (*blue line*), PVP (*pink line*), and after the transfer into IPA (*yellow line*). (Color figure online)

For the transfer procedure, we used NRs stabilized by Na_2_S, whose properties were described before (Gordel et al. 2014). The extinction spectra were recorded before the transfer procedure, at each stage of covering the surface by subsequent polymer layers and after completing the transfer (Fig. 2). The maximum of l-SPR band of freshly synthesized NRs in water was placed at 817 nm, after it was covered by PSS the band was shifted to 835 nm, the covering by PAH caused a shift to 848 nm, and, finally by PVP, to 859 nm. The plasmon redshift occurs in the presence of the polymer layers because of the higher refractive index of the used polymers relative to water. The additional layers surrounding a gold nanorod screen the electron oscillation in the metal, decreasing the plasmon energy (Prodan et al. 2002).When NRs are dispersed in IPA, a significant change is seen in the intensity, location, and half width of the longitudinal surface plasmon band (l-SPR), namely, a red shift of around 100 nm, and the band becomes approximately twice broader.

Electrokinetic potentials were measured in two isotonic buffers, one of pH = 6.24 and another of pH = 7.45. These are high ionic strength media, the total concentration of phosphates being 66.7 mM and NaCl 76 mM. Such high ionic strength results in suppression of electrical double layer, due to low Debye-Hückel ionic atmosphere radius, κ^−1^. The ζ-potential of bare NRs is −4.4 and −32.6 mV at pH 6.24 and 7.45, respectively (Table 1). Adsorption of the PSS layer decreases the potential down to –13.6 mV at pH 6.24, but the ζ-potential at pH 7.45 remains almost unchanged (−31.9 mV). This may be related to the presence of CTAB (de la Cruz et al. 2013), which is present on bare NRs, interaction with kosmotropic phosphate ions (Merk et al. 2014), or reduction of electrical double layer by high ionic strength (Marsalek 2014; Salgin et al. 2012). It has been shown that phosphates interact with primary amines via hydrogen bonding (Irigoyen et al. 2009). This results in no surface charge reversal after adsorption of PAH layer on PSS layer; apparently, this phenomenon is quite common (Caruso and Mohwald 1999; Shenoy et al. 2003; Smith et al. 2004), but usually occurs after adsorption of several layers. After adsorption of PVP, the values of ζ-potential become negative, but their absolute values are remarkably lower. This polymer is weakly charged (no ionic groups present), thus electrical double layer arises from adsorption of ions onto the surface of coated nanoparticles. When the measurements were carried out in IPA, broad peaks of ζ-potential values were observed. Electrical conductivity was sufficient to carry out the measurements (1–2 mS·m^−1^), but the quality of data was much lower than that in aqueous media. Moreover, it is difficult to compare these sets of data, because the ζ-potential is strongly related to the pH of the solution; thus, there is no direct relationship between results for aqueous and IPA media.

**Table 1:** ζ-potential (mV) for studied NRs

Sample name	Experimental conditions	ζ-potential (mV)	Peak half width (mV)
NRs@CTAB	pH = 6.24	−4.4 (±1.7)	4.0 (±0)
	pH = 7.45	−32.6 (±1.1)	4.0 (±0)
NRs@PSS	pH = 6.24	−13.6 (±2.0)	4.0 (±0)
	pH = 7.45	−31.9 (±0.7)	4.0 (±0)
NRs@PAH	pH = 6.24	−13.5 (±1.5)	4.0 (±0)
	pH = 7.45	−32.1 (±1.2)	4.0 (±0)
NRs@PVP	pH = 6.24	−0.8 (±2.9)	4.0 (±0)
	pH = 7.45	−27.3 (±2.4)	4.0 (±0)
NRs IPA	Dielectric constant = 19.23	−1.6 (±6.0)	15.0 (±0)
	Viscosity = 1.96 cP		

**Fig. 3:**
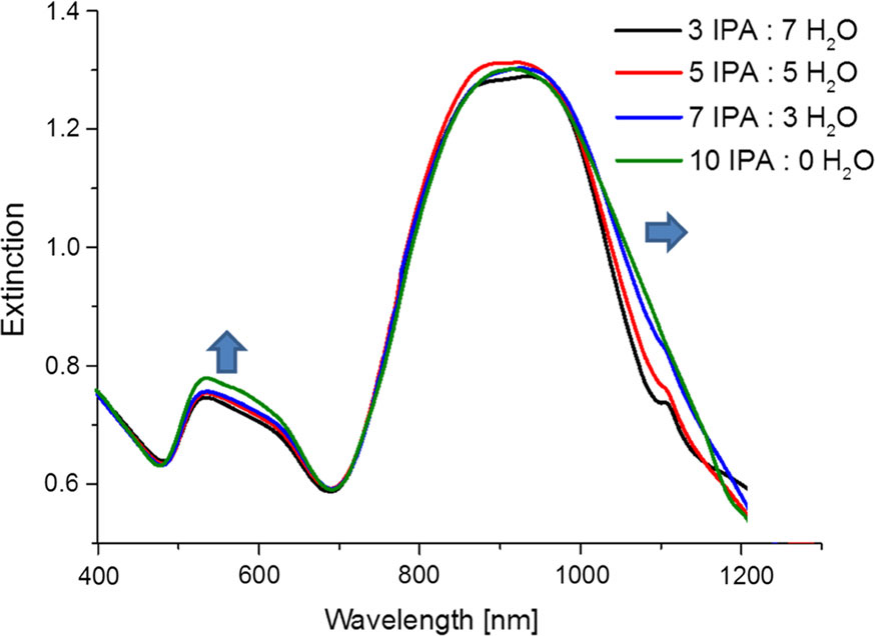
Extinction spectra of NRs suspended in solvent mixtures with a different IPA to H_2_O ratio

**Fig. 4:**
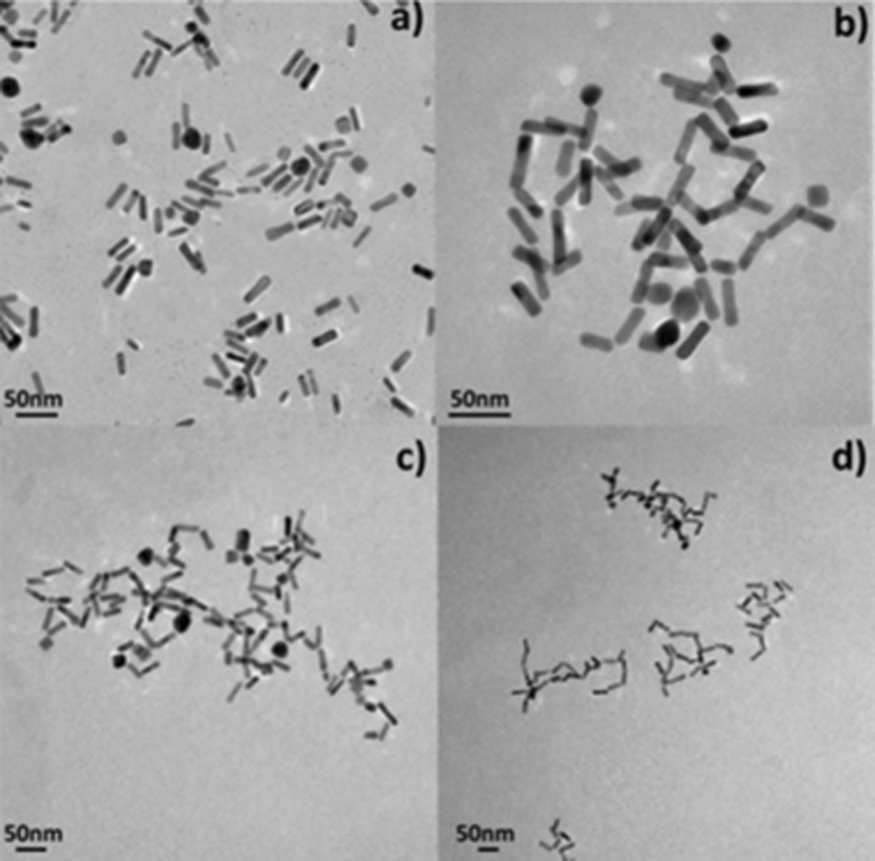
Transmission electron microscope (TEM) pictures of NRs in water (**a**) and in IPA (**b**–**d**). NRs have a tendency to link one to each other by the ends, which is clearly visible in the pictures; on the other hand, this mechanism is not operative in for NRs in water

Additional studies were performed in order to check the change in extinction spectra of NRs occurring when they were redispersed in different IPA to water ratios (Fig. 3). Two modifications were observed on increase of the IPA to H_2_O ratio: an increase in the strength of the t-SPR band and broadening of the long wavelength side of the l-SPR band. Figure 4 shows TEM pictures of the studied samples dried from water (Fig. 4a) and from IPA solvent (Fig. 4b–d). There is a clear tendency for the NRs to form assemblies with preferential end-to-end orientation. As the distances between nanorods are small, coupling of the longitudinal plasmon modes may occur, causing a red shift of the band relative to the original position. Using a dipolar exciton coupling model, the group of El-Sayed (Jain et al. 2006) has explained the shift of the plasmon band upon coupling between plasmon modes. In the end-to-end configuration (J-type dimers), the transition to the higher energy state is forbidden because of the cancelation of the two dipole moments; however, a new band appears at a lower energy with respect to the single nanorod where the interaction between the dipoles is attractive. There is no change in the transverse plasmon band, as the transverse plasmon dipoles are far apart even when the rods touch each other, preventing the interaction at a significant level.

### Spectroscopic characteristics

**Fig. 5:**
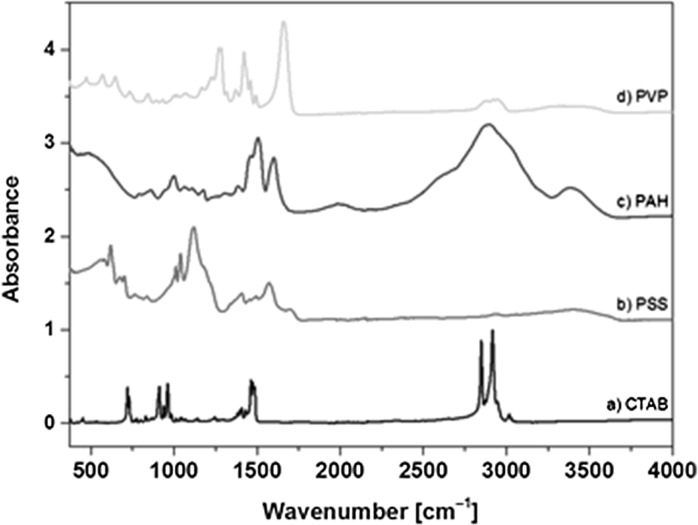
ATR spectrum of pure powdered CTAB (*a*), PSS (*b*), PAH (*c*), and PVP (*d*) in the 370–4000 cm^−1^ range

**Fig. 6:**
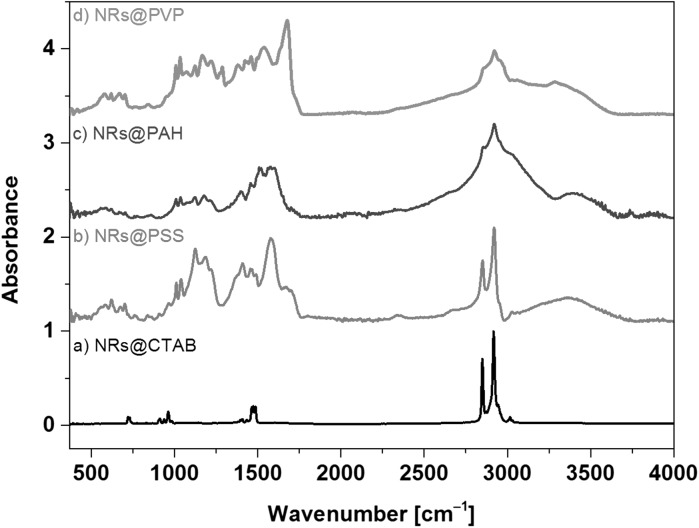
ATR spectrum of gold nanorods at each step of preparation: CTAB stabilized gold NRs (*a*), gold NRs coated with PSS (*b*) and PAH (*c*) and gold NRs coated with PVP (*d*) in the 370–4000 cm^−1^ range

Modification of gold NRs surfaces was monitored by ATR spectra at each step of the process (after adding CTAB, PSS, PAH and PVP to gold NRs, respectively) and the spectra were compared with those of powdered CTAB, PSS, PAH, and PVP as shown in Figs. 5 and 6.

No differences are observed between ATR spectra of pure CTAB and gold NRs coated by CTAB (Figs. 5a, 6a) which proves that CTAB molecules are connected to the NRs only by electrostatic attractions (Gui and Cui 2012). The most characteristic intense bands of CTAB molecules appear at 2916 and 2849 cm^−1^ (anti-symmetrical and symmetrical stretching aliphatic –CH bands, respectively) (Garabagiu 2013), 961 (C–N stretching), 911 (out-of-plane wagging –CH_2_), and 719 cm^−1^ (in-plane deformation rocking –CH_2_) (Wang and Wang 2010). Positions of these bands remain essentially the same in NRs@CTAB (Fig. 5a), small differences in the positions of the peak are likely due to the presence of surface-bonded CTAB molecules (Sau and Murphy 2005).

In the next step, PSS molecules are added to NRs@CTAB in water solution. The characteristic bands of powdered PSS appear at 1573 (C=C stretching), 1406 (O=S=O antisymmetric stretching) (Shim and Weiss 2005), 1183 (${\text{SO}}_{3}^{ - }$ asymmetric stretching) (Pitia et al. 2011), 1118 (O=S=O symmetric stretching), 1039 (${\text{SO}}_{3}^{ - }$ symmetric stretching), 1010 (C–S symmetric stretching), and 836 cm^−1^ (out-of-plane bending C–H vibration for *para*-substituted phenyl ring) (Brijmohan et al. 2005). After adding PSS to NRs@CTAB solution there is evidence in the ATR spectrum that both PSS and CTAB molecules are connected to gold NRs (Fig. 6b) as bands at 2920 and 2851 cm^−1^ from CTAB molecules are observed. Similar relative intensities of the most characteristic bands of CTAB and PSS confirm that the concentration of both compounds is comparable. However, the position of an antisymmetric stretching S=O band at 1124 cm^−1^ in NRs@PSS spectrum is 6 cm^−1^ shifted in comparison with powdered PSS. Additionally, a new band at 1185 cm^−1^ appears (Fig. 6b). These changes can be connected with weak intermolecular interactions between PSS and CTAB in which S=O groups are involved, or with electrostatic repulsion between polymer layers (Maurdev et al. 2002). Thus, weak C–H···O hydrogen bonds between methyl group from CTAB and oxygen atoms in sulphonate group from PSS may stabilize both molecules on the gold NRs surface. This result indicates that there is only weak attractive electrostatic or/and electrosteric interaction between both molecules and the surface (Muir et al. 2001).

The characteristic bands of powdered PAH (Fig. 5c) are located at 3382 (stretching antisymmetric N–H), 2897 (stretching –CH_2_), 1601, and 1506 cm^−1^ (in-plane-bending –NH). In the ATR spectrum of NRs@PAH, CTAB bands at 2292 and 2853 cm^−1^ are almost covered by a wide and strong one at 2897 cm^−1^ (Fig. 6c). Wavenumber positions of overlapped bands from PAH and PSS in the 1400–1600 cm^−1^ range do not show significant changes in comparison with the pure compounds.

**Fig. 7:**
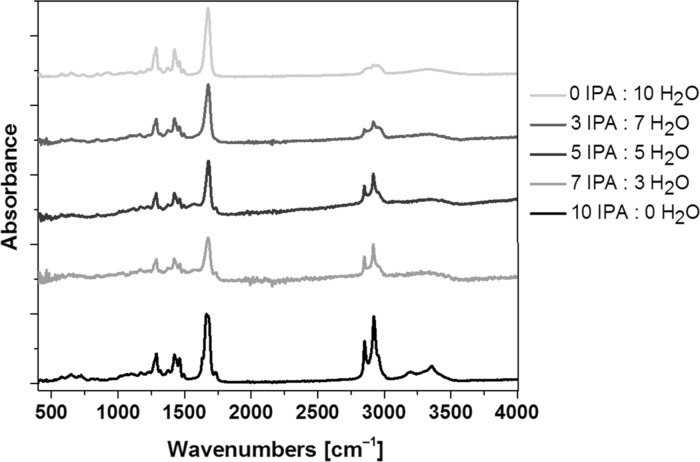
ATR spectra of NRs suspended in solvent mixtures with a different IPA to H_2_O ratio in the 370–4000 cm^−1^ range

The most characteristic band of powdered PVP is carbonyl stretching C=O at 1658 cm^−1^ (Fig. 5d). In the case of gold NRs ATR spectrum, this stretching band is shifted to 1677 cm^−1^ (Fig. 6d). This band position depends on the concentration of water molecules (Borodko et al. 2006). This band is observed, respectively, at 1658 cm^−1^ (powdered PVP), 1664 cm^−1^ (the NRs in IPA solution), and at 1675 cm^−1^ (the NRs in H_2_O/IPA mixtures at any ratio of the solvents, Fig. 7). However, there are no significant changes in this band relative intensity or position in the spectra measured at a different ratio of IPA and H_2_O, which suggests that no significant interactions are present between the solvents (IPA or water) and PVP molecules.

The spectrum of gold NRs@PVP reveals significant concentrations of each type of polymer molecules in the last step of NRs coating as sharp bands from CTAB (at 2922 cm^−1^) and from PAH (1539 cm^−1^) appear in the spectrum. The intensities of bands assigned to stretching C–N and scissoring bending –CH_2_ and wagging bending –CH_2_ vibrations (Borodko et al. 2006) at 1459, 1421, 1283, and 1270 cm^−1^ in powdered PVP decrease strongly in NRs@PVP ATR spectrum. This suggests that the main alkyl chain in this polymer is attached to other molecules on gold NRs surface.

The N–H stretching band observed in NRs@PAH at 3382 is 96 cm^−1^ red-shifted to 3286 cm^−1^ in NRs coated with PVP. It is possible that the oxygen atom from the carbonyl group participates in weak N–H···O hydrogen bonds between carbonyl and amino groups from PVP and PAH, respectively. The N–H in-plane deformation band at 1539 cm^−1^ in NRs@PAH ATR spectrum is 33 cm^−1^ blue-shifted in comparison with the same band in powdered PAH at 1506 cm^−1^.

The above results strongly suggest that the end-to-end assembly of gold NRs is due to weak hydrogen bonds between PAH and PVP at the ends of the NRs. The proposed mechanism of this process is presented in Fig. 1. PVP is a neutral polymer with a strong electron-acceptor C=O group; however, PAH is a cationic polymer with electron-acceptor –NH_3_^+^ group. We suggest that these polymers bind by weak hydrogen bonds between PVP and PAH polymer. Although the IPA environment plays a significant role in end-to-end assembly of the NRs, the IPA molecules do not enter directly into the interactions between the polymer chains.

### Theoretical simulation

**Fig. 8:**
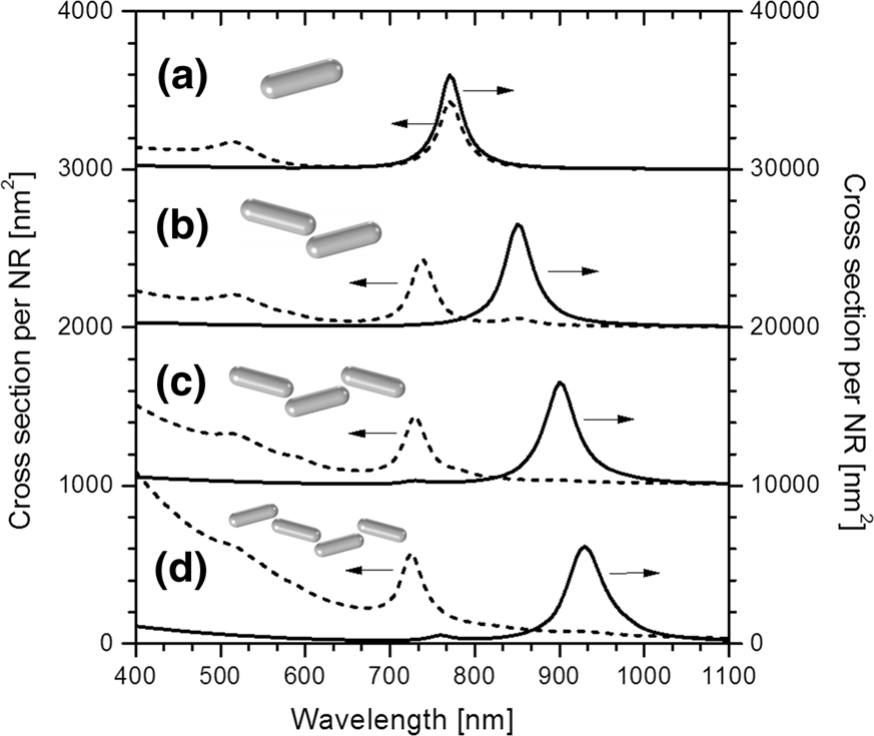
Extinction spectra of NR chains of various lengths, calculated using COMSOL: (*a*) single NR, (*b*) pair of NRs, (*c*) three NRs, and (*d*) four NRs, oriented end-to-end.* Solid lines* show the extinction cross sections per NR at incident polarization parallel to the chains, and correspond to the vertical axis on the right-hand side of the plot, whereas* dashed lines* are associated with cross sections per NR at polarization perpendicular to the chain, and the corresponding values are displayed on the *left vertical axis*. The *insets* show the geometries of the NR chain models

**Fig. 9:**
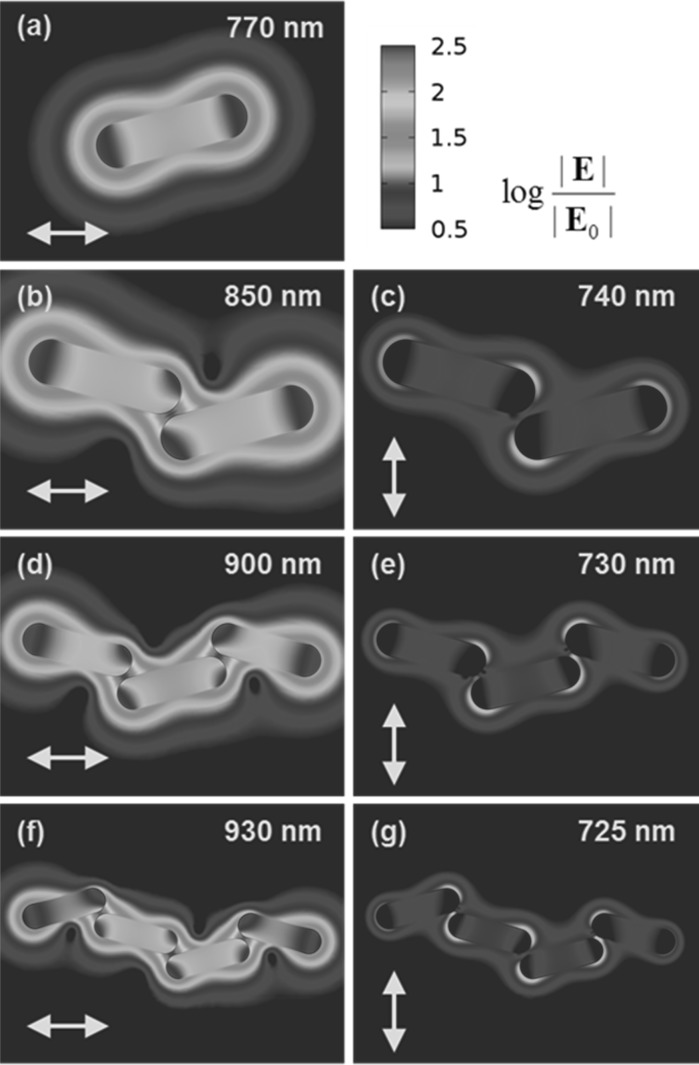
Spatial distribution of the logarithm of the electric field enhancement around: **a** single NR, and chains composed of **b**, **c** two, **d**, **e** three, and **f**, **g** four NRs. Images on the *left* (**b**, **d**, **f**) correspond to the bonding plasmon modes and incident polarization parallel to the chains, whereas the images on the *right* (**c**, **e**, **g**) show the electric field distribution at the anti-bonding plasmon resonances under polarization perpendicular to the chains. The *color* scale next to the top image (**a**) is common for all images

Extinction spectra, calculated in COMSOL, are plotted in Fig. 8, and the electric field distribution corresponding to particular plasmon modes is presented in logarithmic scale in Fig. 9. In the case of a single NR, the extinction spectrum (Fig. 8a) features two localized surface plasmon modes: the longitudinal mode (*λ* = 770 nm, close to the experimental value 820 nm) and the transverse mode (*λ* = 515 nm). When the incident polarization is set to ±75° with respect to the long axis of the NR, these two modes appear simultaneously and with comparable cross sections (~430 and ~170 nm^2^, respectively); however, at ±15°, the longitudinal mode becomes dominant, appearing with a cross section higher by more than an order of magnitude (~6000 nm^2^). Local field enhancement at the longitudinal resonance exceeds a factor of 50 at the NR tips (Fig. 9a), contrary to very weak transverse resonance that shows a field enhancement factor of only 3 (not presented).

The above plasmonic properties change dramatically when two NRs are set together end-to-end at a very short distance. The longitudinal mode hybridizes into two separate plasmon modes: the low-energy bonding mode (*λ* = 850 nm) and the high-energy anti-bonding mode (*λ* = 740 nm) (Fig. 8b). The latter one is more apparent at incident polarization perpendicular to the chain (the orientation and arrangement of the NRs are such that the gap axis does not coincide exactly with the chain axis, enabling the interactions even at incident polarization perpendicular to the chain). This plasmon mode interacts rather weakly with the incident light, resulting in a cross section per NR equal to ~430 nm^2^ and local field enhancement of around 15-fold. In contrast, the cross section per NR at the bonding resonance reaches maximal value of ~6500 nm^2^ at polarization parallel to the chain. The electric field is strongly localized in the gap, where the enhancement exceeds a factor of 300. Two NRs are bound together by a strong interaction that makes them behaving like a single entity—a longer NR with a red-shifted longitudinal plasmon resonance, which is visible in Fig. 9b. The situation is opposite at the anti-bonding resonance, where the strong repulsion between the surface charges of the two NRs pushes the electric field away from the gap region, damping the individual surface charge oscillations of the NRs and reducing the local electric field enhancement (Fig. 9c).

The above hybridization effect is even stronger when longer chains are formed. In the case of a chain made of three NRs (Fig. 8c), the bonding resonance is further red-shifted up to *λ* = 900 nm, and the “hot-spots” inside the gaps exhibit field enhancements exceeding a factor of 350 (Fig. 9d). The anti-bonding mode is slightly blue-shifted down to 730 nm, and the electric field distribution is similar to that of a chain of two NRs, with the maximum enhancement of around 15-fold (Fig. 8e). Extinction spectrum of a chain consisting of four NRs (Fig. 9d) confirms the above trend, showing a further red shift of the bonding mode up to 930 nm and a blue shift of the anti-bonding mode down to 725 nm. Also the field distribution (Fig. 9f–g) features similar behavior to that of a chain composed of three NRs.

One may expect that the addition of additional segments will cause further red shifting of the bonding mode. Moreover, in analogy to the molecular orbitals, the plasmonic hybridization scheme predicts the appearance of a given number of additional energy levels determined by the number of NRs participating in the hybridization. However, because of the end-to-end arrangement within the chains, such additional modes are rather weak, so that the observable extinction spectra are dominated by the main bonding and anti-bonding resonances. On the other hand, very long chains of NRs will exhibit additional higher order modes corresponding to plasmonic standing waves, formed by propagating plasmon polariton modes guided along the NR chains (Gui and Cui 2012). As a result, the extinction spectrum measured on a liquid dispersion of NR chains of various lengths will be covered by a distribution of resonances. The overlap of these resonances is probably the main origin of the broad, red-shifted extinction band measured in the IPA solution of NRs, covering the spectral region 700–1100 nm.

## Conclusions

We investigated the process of transferring the NRs from water into isopropanol. We report self-assembly of the NRs based on weak non-covalent interactions that can be easily adopted for fabrication of optical materials for biological sensing. The suggested mechanism of coating the nanorod surface by polymers is based on the weak attractive electrostatic and intermolecular interactions between molecules. The CTAB molecules create a hydrophobic microenvironment near the surface of the NRs. By using a Layer-by-Layer Method, the NRs were transferred from water into IPA. In non-polar environments, very weak N–H···O hydrogen bonds occur between amino groups of PAH and oxygen atoms from carbonyl groups in PVP. This is evidenced by ATR spectra where the characteristic stretching as well as in-plane deformation band of the NH oscillator is red-shifted (by about 96 cm^−1^) and blue-shifted (by about 33 cm^−1^), respectively. We were able to characterize the type of interaction between the polymers used as ligands during the transfer procedure and their contribution to the final stage, when the NRs were dispersed in IPA solution. The tendency of the NRs dispersed in IPA solution to self-assemble into gold nanochains consisting of a various number of NRs causes a red shift of the l-SPR and broadening of the near-infrared extinction band due to overlap of multiple plasmon resonances at various wavelengths covering the spectral region 700–1100 nm. Individual self-assembled nanostructures show strong light polarization-dependent properties. The electric field localized in the gap between NRs is estimated to be enhanced over 350-fold.
